# Female Germ Cell Development in Chickens and Humans: The Chicken Oocyte Enriched Genes Convergent and Divergent with the Human Oocyte

**DOI:** 10.3390/ijms231911412

**Published:** 2022-09-27

**Authors:** Deivendran Rengaraj, Jae Yong Han

**Affiliations:** Department of Agricultural Biotechnology, Research Institute of Agriculture and Life Sciences, Seoul National University, 1 Gwanak-ro, Gwanak-gu, Seoul 08826, Korea

**Keywords:** germ cell, oocyte, gene expression, gene conservation, chicken, human

## Abstract

The development of germ cells and other physiological events in the differentiated ovary of humans are highly conserved with several mammalian species, except for the differences in timing. However, comparative knowledge on this topic is very scarce with respect to humans and lower vertebrates, such as chickens. In chickens, female germ cells enter into meiosis around embryonic day (E) 15.5 and are arrested in meiotic prophase I as primary oocytes. The oocytes arrested in meiosis I are accumulated in germ-cell cysts; shortly after hatching, they are enclosed by flattened granulosa cells in order to form primordial follicles. In humans, the process of meiotic recombination in female germ cells begins in the 10–11th week of gestation, and primordial follicles are formed at around week 20. In this review, we comprehensively elucidate both the conservation and the species-specific differences between chickens and humans with respect to germ cell, oocyte, and follicle development. Importantly, we provide functional insights into a set of chicken oocyte enriched genes (from E16 to 1 week post-hatch) that show convergent and divergent expression patterns with respect to the human oocyte (from week 11 to 26).

## 1. Introduction

The principal female germ cells, oocytes, are developed from primordial germ cells (PGCs) that are usually specified in an extragonadal region during early embryogenesis. The specification of PGCs was reported to take place via either an epigenesis mode (particularly in Mammalia and Caudata) or an inherited mode (in Aves, Anura, Teleostei, and Insecta) in animals [1]. Irrespective of these specification modes, the specified PGCs migrate to the gonads and simultaneously undergo epigenetic reprogramming, including genome-wide DNA demethylation and dynamic changes in histone modifications [2,3,4]. Fertility functions, including germ cell development in females and males, are essential for producing healthy offspring [5]. Particularly in the female sex, late gonadal PGCs, or oogonia, are properly entered into meiosis and arrested in meiotic prophase I as oocytes; the oocytes are also surrounded by the granulosa and theca cell layers during ovarian follicular development [6,7,8,9]. In addition, the oocytes store several RNA transcripts, proteins, and cytoplasmic organelles that are passed to offspring and play a critical role during zygotic genome activation [10,11]. Moreover, it was reported that a large number of genes are differentially expressed and play a critical role during oocyte meiosis [12,13,14]. The development of PGCs, oocytes, and ovarian follicles involves highly conserved processes among mammalian species and non-mammalian vertebrates. Considering the greater evolutionary distance between chickens and humans compared to the differences between other mammals and humans, a comparative study at the transcriptome level related to the differences in the development of female germ cells between these species is necessary. Such a study is helpful for determining whether or not the regulatory system of germ cells is preserved between species, which is a question that has great significance in the field of germ cell research. Therefore, we address two main issues in this review: the first is the transcriptome-based conservation of female germ cell development in chickens and humans; the second involves functional insights with respect to chicken oocyte enriched genes that are convergent and divergent with the human oocyte.

## 2. Development of Germ Cells, Oocytes, and Ovarian Follicles in Chickens

**Figure 1:**
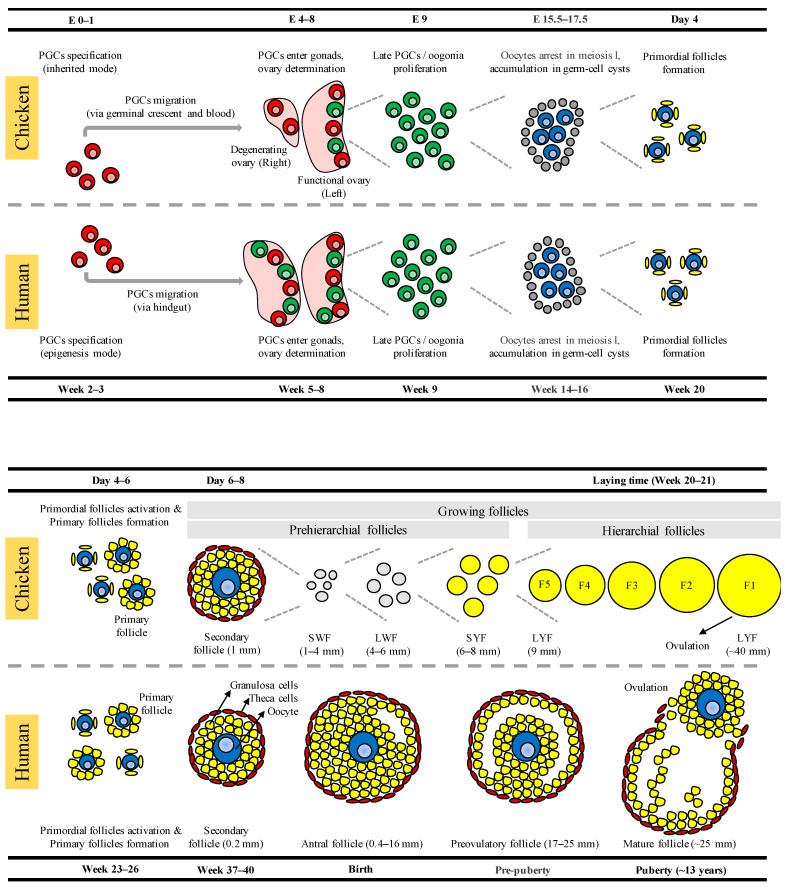
Schematic comparison of developing germ cells, oocytes, and ovarian follicles in chickens and humans. The primordial germ cells (PGCs) are specified by the inherited mode in chickens and by the epigenesis mode in humans. After specification, PGCs migrate to the gonads; this occurs via the germinal crescent and blood circulation in chickens and via the hindgut in humans. Further physiological events in the differentiated ovary, including oogonia proliferation and entry into meiosis, oocyte arrest in meiotic prophase I, the accumulation of oocytes in germ-cell cysts, and the formation of early follicles, are all essentially the same in both chickens and humans, except the differences in timing. In contrast, late folliculogenesis is meaningfully different between chickens and humans. SWF: small white follicles; LWF: large white follicles; SYF: small yellow follicles; LYF: large yellow follicles.

In chickens (Figure 1), the precursor cells of oocytes, which are called PGCs, are specified by the inherited mode (also referred to as preformation mode). In this mode, a set of RNAs, proteins, and selected energy-rich mitochondria is stored in the germ plasm of mature oocytes and are exclusively allocated to prospective PGCs when the zygote undergoes cleavage and cellularization [1,15]. After specification, the PGCs are mainly located in the central region of the embryo during chicken intrauterine embryonic development. During in ovo embryonic development, PGCs polarize first and move passively toward the anterior region by the morphogenetic movement of the embryo. In the anterior part, PGCs are incorporated into the semi-circular-shaped germinal crescent region. At about embryonic day 2 (E2.0), PGCs enter into the lumina of blood vessels and remain in blood circulation. The circulating PGCs enter into the genital ridge (i.e., the future gonadal region) at around E3.0–E3.5 [15,16,17,18]. Furthermore, it was reported that the genital ridge cells secrete a chemoattractant stromal cell-derived factor 1 (SDF1), which is received by PGC G protein-coupled receptor C-X-C motif chemokine receptor 4 (CXCR4) during the migration of PGCs toward the genital ridge [19]. Additionally, the migrating PGCs undergo genome-wide DNA demethylation, and de novo DNA methylation is then established in PGCs entering the gonads [2,4].

In chicken gonads, PGCs undergo dynamic proliferation and differentiate into oogonia (at about E8.0) shortly after the female sex of the gonads is determined, and the oogonia continue dynamic proliferation until the first meiotic arrest occurs in the female [20,21,22]. Asymmetric gonadal development, in which only the left gonad of the female becomes a functional ovary, is very common in chickens and other avian species. In contrast, the right gonad undergoes degeneration [23]. It was reported in chickens that the retinoic acid (RA) produced by the mesonephros promotes meiosis initiation in oocytes by triggering the expression of the pre-meiotic marker that is stimulated by retinoic acid 8 (*STRA8*), as well as the subsequent expression of synaptonemal complex proteins (SYCPs), such as *SYCP1*, *SYCP2*, and *SYCP3* [20,24]. The cells that entered into meiosis are arrested in meiotic prophase I as oocytes (termed primary oocytes) from E15.5, with the subsequent upregulation of meiotic prophase I markers, such as SPO11, initiator of meiotic double-stranded breaks (*SPO11*); RAD21 cohesin complex component like 1 (*RAD21L1*); and piwi like RNA-mediated gene silencing 1 (*PIWIL1*) [20,21,25]. From E17, the chicken ovaries show a composite population of oocytes at different stages of meiotic prophase I, and the maximum amount of pachytene stage oocytes was, in a previous work, found around the time of hatching [26]. Although the process is not clear in chickens, meiotic-arrested oocytes accumulated in germ-cell cysts have been reported at least 1 day post-hatch and 4 weeks post-hatch in the ovaries [2,21,27]. 

Primordial follicles are first-stage follicles formed in the ovary, and these follicles remain in a dormant state until the ovarian cyclic activity induces the growth of selected follicles [7]. Along with the sharp increase in follicle-stimulating hormone receptors (FSHRs), the primordial follicles start to develop within 4 days of hatching by the breakdown of germ-cell cysts and the enclosure of oocytes by a single layer of flattened pre-granulosa cells [28]. In the primary follicle, the oocyte is enclosed by differentiated cuboidal granulosa cells. In contrast, in the secondary follicle, the oocyte is enclosed by granulosa cells and theca cells [6,7]. The growing follicles appear from 6 days after hatching [28]. At this time, oocytes store all of the components (such as RNAs, proteins, and cytoplasmic organelles) that are later required for zygotic genome activation [11]. The growing follicles, which are notably without antrum or follicular fluid, naturally protrude on the surface of the ovary and are classified based on their size and developmental stage, including prehierarchical growing follicles (small white follicles (SWF, 1–4 mm), large white follicles (LWF, 4–6 mm), and small yellow follicles (SYF, 6–8 mm)) and preovulatory hierarchical follicles (large yellow follicles (LYF, 9–40 mm)) [6,7,29,30]. A mature oocyte from the largest yolk-filled hierarchical follicle is released into the infundibulum of the chicken oviduct. Here, the ovulated ovum, or egg, is surrounded only by the perivitelline layer (equivalent to the mammalian zona pellucida) and not by the granulosa cell layer [8,31]. During follicular development, the oocytes are continuously arrested in meiotic prophase I; they resume from meiosis I when oocyte growth is fully achieved (i.e., a few hours before ovulation) but are again arrested in the metaphase of meiosis II until ovulation, which is a phenomenon similar to what occurs in mammalian oocytes [8,9,11].

## 3. Development of Germ Cells, Oocytes, and Ovarian Follicles in Humans

In humans (Figure 1), the PGCs are specified by epigenesis mode (also referred to as induction mode). In this mode, PGCs are specified in the early post-implantation embryo by bone morphogenetic protein 4 (BMP4) and Wnt family member 3 (WNT3) signaling from extra-embryonic tissues, which is the same as what occurs in mice [32]. However, the core regulatory network induced by these signals in a few pluripotent epiblast cells for germ cell fate determination differs between mice and humans. The core regulatory network in humans includes four transcription factors, namely SRY-box transcription factor 17 (*SOX17*), PR/SET domain 1 (*PRDM1*), *PRDM14*, and transcription factor AP-2 gamma (*TFAP2C*), that are necessary for upregulating germ cells and pluripotency genes while repressing WNT signaling and somatic markers [32,33,34,35]. The human PGCs are established around the time of gastrulation (week 2–3), and the specified PGCs are localized near the yolk sac wall, close to the allantois, at week 4. Then, PGCs migrate through the hindgut and colonize the developing genital ridge by early week 6. In humans, the migrating PGCs also exhibit hallmark properties, including genome-wide DNA demethylation, dynamic changes in histone modifications, and imprint erasure, as in mice PGCs [32]. Additionally, the loss of DNA methylation and repressive H3K27me3 at the inactivated X chromosome leads to X chromosome reactivation in female PGCs, ensuring that each oocyte contains an active X chromosome [32,36]. Gonadal PGCs remain proliferative and rapid, particularly in female gonads, until around week 9. This week is a critical time point in human female germ cell development because the cells are considered late PGCs or oogonia; however, the oogonia remain hypomethylated until birth [3,32,33].

The initiation of oocyte meiosis by the mesonephros secreting RA that triggers the expression of *STRA8*, as described above, is the same process that takes place in the human fetal ovary. In addition, processes related to oocyte/follicular development in humans are highly conserved with mice, which is a well-studied animal in this regard, and with a few other mammalian species, such as cows and pigs, with some differences involving timing and genes/proteins [37]. Specifically, the process of meiotic recombination in human female germ cells begins asynchronously in week 10–11, and *PRDM9* has been shown to be a major determinant of meiotic recombination hotspots [38,39]. The cells entered into meiosis are arrested in meiotic prophase I as primary oocytes with decondensed chromatin (also called dictyate-stage arrest) in week 14–15 [38]. The Balbiani body (Bb) is a prominent feature in the oocytes of many diverse organisms. It is an aggregate of proteins, germ plasm, mRNA, membrane-bound organelles, and mitochondria at one pole of the oocyte nucleus [1]. It was observed that the Bb of human primary oocytes contains more enriched mitochondria than that of mouse primary oocytes, which contain no or only a few mitochondria [40,41].

The primordial follicles start to develop around week 20 by the breakdown of germ-cell cysts and the enclosure of oocytes by flattened pre-granulosa cells [3]. Furthermore, transcriptional activity in the oocyte appears discontinuous throughout folliculogenesis in humans, which is similar to what occurs in mice, cows, and pigs, and transcription is hardly detectable in the oocyte of primordial follicles [37]. These dormant primordial follicles, termed the ovarian reserve, are the principal marker of female fertility. Among several hundred thousand primordial follicles present in the fetal ovary, only a small number of primordial follicles are activated shortly before birth to develop and enter the growing pool of follicles; these include primary follicles, secondary follicles (preantral stages), antral follicles, and preovulatory follicles [42,43]. Moreover, excessive activation of primordial follicles causes the development of premature ovarian insufficiency, characterized by the loss of ovarian follicles and menopause before 40 years old [43,44]. In preantral follicle development, the oocytes grow in size and reach their maximum size when the diameter of secondary follicles reaches about 0.2 mm; however, the weight of the thecal tissue, the number of granulosa cells, and the volume of the antral fluid increase with respect to the diameter of healthy antral follicles (0.4–16 mm) and preovulatory follicles (17–25 mm) [45,46]. When menstrual cycles begin after sexual maturity in response to gonadotropin stimulation, fully grown primary oocytes resume meiosis I cell division, extrude first polar bodies, and form secondary oocytes. The ovulated secondary oocytes (referred to as the egg–cumulus complex leaving the mature follicle) complete meiosis II cell division only after fertilization, which is a phenomenon that is similar in humans and most mammalian species [3,47].

## 4. Ovarian Follicles at Young and Old Ages

The ovary is the principal female reproductive organ, in which follicles develop and the secretion of female sex hormones (estrogen and progesterone) takes place. In chickens, studies on the ovaries of both young and old birds are more limited than those studying birds of adult age. A recent study analyzed the ovaries of post-hatch chickens at ages ranging from 1 to 16 weeks old and revealed that ovary features are closely related to follicular developmental stage. Notably, follicles are in a very slow-growth phase from 1 to 3 weeks of age, entering a fast-growth phase from 4 to 16 weeks and bulging the ovarian tissue. In addition, atretic follicles at various regression stages are observable from 3 weeks of age [48]. The onset of egg-laying time in chickens occurs at about 20–21 weeks. The egg production rate increases 6 to 8 weeks after the onset of egg laying, and peak rate is maintained for a few months. The egg production rate normally decreases in older chickens (150–200 weeks), and they lay fewer but larger eggs than younger chickens (30–40 weeks). This phenomenon in older chickens is connected with a reduced rate of follicle recruitment for rapid growth and a lengthy period of follicular growth and development [49]. Several factors, including the environmental conditions and nutrition supply, influence the decrease in egg production in laying chickens [50]. However, oocyte apoptosis, a failure to maintain the ovarian reserve due to the excessive activation of primordial follicles for rapid growth, elevated levels of reactive oxygen species (an oxidative stress condition), the formation of a high number of atretic follicles, and a decline in liver function are factors primarily associated with the gradual decrease in, and even cessation of, egg production in older chickens [51,52,53].

In humans, preantral ovarian follicles enter the early antral stage and undergo subsequent stages of development via the accumulation of antral fluid and the proliferation of granulosa cells and theca interna cells [54]. A significant proportion of follicles present at the time of birth become atretic, with fewer than 1% undergoing ovulation [46]. Atresia represents the breakdown of ovarian follicles, and it is necessary for it to occur throughout life for women to maintain a healthy reproductive system [55]. It was demonstrated that collagen content increased, while levels of hyaluronan decreased, in the ovaries of humans between the ages of 11–20 and older than 51. These age-dependent changes were associated with ovarian stiffness and may impact follicle development and oocyte quality [56]. The onset of ovulation time in humans is about 13 years old; in contrast to chickens, humans usually ovulate one egg per menstrual cycle (which includes the follicular phase, the ovulation phase, and the luteal phase) from a fully mature follicle. Furthermore, the number of selectable follicles per ovary and the number of all classes of growing follicles decreases significantly in humans over the age of 40 when compared to younger women aged 19–30 [54]. In fact, a woman’s capacity to reproduce decreases, after a peak of efficiency in her 20s, due to various age-dependent cellular and molecular changes in the ovarian follicles [57]. The age-related decrease in the number of follicles dictates the onset of cycle irregularity and the final cessation of the menstrual cycle [58]. The final menstrual period (so-called natural menopause) occurs at a mean age of 51 in women, when the decrease in follicles reaches about 1000 from a peak of two million at birth; however, the age range for the onset of menopause varies between 40 and 60 in some women [42,58,59]. 

## 5. Transcriptome-Based Conservation of Female Germ Cell Development in Chickens and Humans

The conservation of germ cell development, including oocyte development and folliculogenesis, in humans and several other mammalian species, such as mice, rats, cows, pigs, and monkeys, has been reported in various earlier studies [3,37,60,61]. Despite some differences in timing and the mechanism of gene and protein expression, the conservation of germ cell development between those species is quite reasonable because they are all from the same vertebrate class. Studies elucidating the conservation of germ cell development between humans and chickens, which are a lower vertebrate, are very rare and disconnected [62]. In a recent paper, chicken germ cell dynamics were determined using single-cell RNA sequencing (scRNA-seq), and the development of chicken and human germ cells during embryogenesis was compared [25]. As a result of analyzing the chicken female germ cells from E2.5 to 1 week post-hatch, five stages (female stage 1 (fS1)—female stage 5 (fS5)) were defined for the chicken female germ cells on the basis of cluster-specific signature scores for the Kyoto encyclopedia of genes and genomes (KEGG) pathways [25]. Additionally, based on the activated signature scores and the associated developmental time points for each stage, the chicken female germ cell stages were annotated as follows: migration to differentiating PGCs (fS1, enriched for cells at E2.5, E6, and E8); mitotic to RA-responsive oogonia (fS2, enriched for cells at E12); RA-responsive to meiotic-arrest oocytes (fS3, enriched for cells at E16 and at hatch); meiotic-arrest to primordial follicular oocytes (fS4, enriched for cells at hatch and at 1 week); and primordial follicular to growing follicular oocytes/apoptotic oocytes (fS5, enriched for cells at 1 week) [20,22,24,25,28].

Based on the available scRNA-seq data for human fetal germ cells (FGCs) and marker gene expression, human female FGCs from week 5 to week 26 after fertilization were divided into four stages, including the mitotic stage (fS1, week 5–26), the RA-responsive stage (fS2, week 11–26), the meiotic stage (fS3, week 14–26), and the oogenesis stage (fS4, week 18–26) [25,63]. Obviously, the length of time required for embryonic development in humans is much longer (268 days median time from ovulation to birth) [64] than the length of time required for embryonic development in chickens (22 days from ovulation to hatching) [65,66]. In addition, due to asynchronous and heterogeneous types of germ cell development in both species, their germ cells were mapped to a wide range of developmental stages: i.e., human fS1 to chicken fS1; human fS2 to chicken fS2–fS4; human fS3–fS4 to chicken fS4 [25].

## 6. Chicken Oocyte Expressed Genes Convergent with the Human Oocyte

An earlier study compared the developmental trajectories of human FGCs with chicken germ cells by inspecting expression dynamics in each species. As a result, the authors identified 119 orthologs dynamically expressed along the human (week 5 to 26) or chicken (E2.5 to 1 week) developmental trajectories in females. Of these orthologs, 83 were convergent genes (evolutionarily conserved), and 36 were divergent (species-specific) genes [25]. 

**Figure 2:**
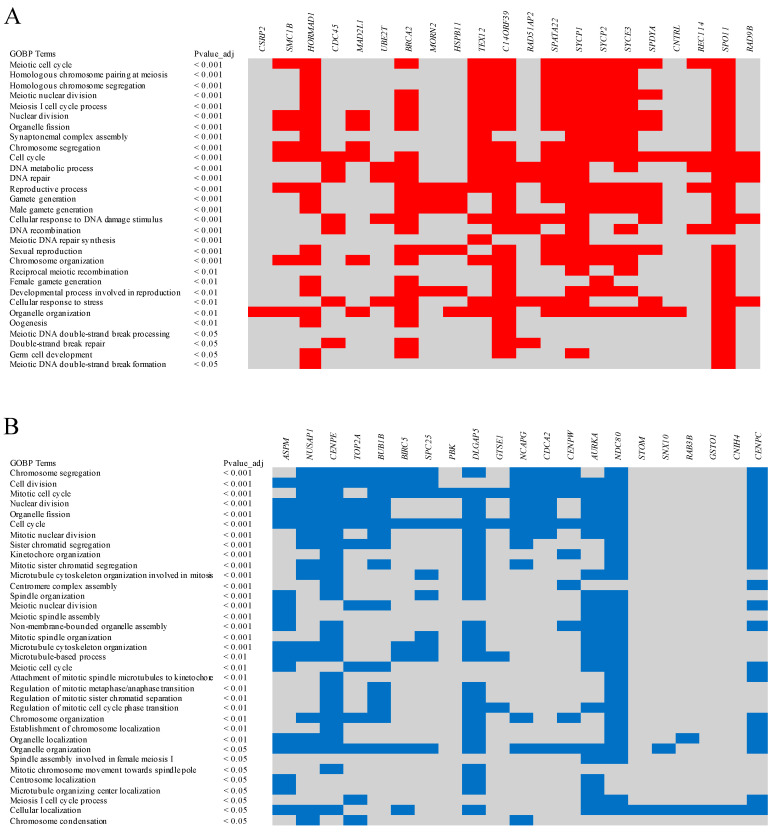
Biological processes of the genes reviewed in this article. (**A**) Biological processes of the chicken oocyte enriched genes that are convergent with the human oocyte. (**B**) Biological processes of the chicken oocyte enriched genes that are divergent from the human oocyte. The involvement of convergent and divergent genes in the biological processes is emphasized in red and blue, respectively.

Hereafter, we study, in particular, the genes enriched in the chicken female germ cells of stages fS3-fS4 (at E16—1 week) because the primary oocytes arrested in meiotic prophase I and accumulated in germ-cell cysts or enclosed by flattened granulosa cells were identified at these time points [20,27,28]. As shown in an earlier study [25], about 29 genes were enriched in the chicken oocytes at E16—1 week and showed convergent expression patterns with human female FGCs (RA-responsive, meiotic, and oogenesis stage cells at week 11–26) (Table 1). The gene ontology biological processes (GOBPs) of all convergent genes were observed using g:Profiler with a g:SCS threshold of 0.05 [67]. Among the convergent genes, at least 21 genes, namely *CSRP2*, *SMC1B*, *HORMAD1*, *CDC45*, *MAD2L1*, *UBE2T*, *BRCA2*, *MORN2*, *HSPB11*, *TEX12*, *C14ORF39*, *RAD51AP2*, *SPATA22*, *SYCP1*, *SYCP2*, *SYCE3*, *SPDYA*, *CNTRL*, *REC114*, *SPO11,* and *RAD9B*, were repeatedly identified in several biological processes related to germ cell development, oocyte development, and meiosis (Figure 2A). Most of the top biological processes were related to meiotic events, such as the meiotic cell cycle, homologous chromosome pairing, homologous chromosome segregation, meiotic nuclear division, and synaptonemal complex assembly.

Male and female germ cells undergo one round of meiotic cell division during their development to reduce the number of complete sets of chromosomes (ploidy), thereby maintaining the ploidy of the species after fertilization [12]. However, the female germ cells are arrested for a longer time in meiotic prophase I as they enter into meiosis much earlier than the male germ cells. Several genes and non-coding RNA transcripts are exclusively expressed and/or developmentally regulated in order to play a critical role during oocyte meiosis [12,13,14]. For instance, with respect to the above convergent genes, *CDC45* plays a critical role in DNA replication to ensure that chromosomal DNA is replicated only once per cell cycle [68]. DNA replication in the S phase is important before the cells enter meiotic prophase I. *SPDYA* is also a cell cycle gene preferentially expressed in the G1/S phase and plays a role in cell cycle progression, including oocyte maturation [69]. The *SPO11*-dependent introduction of DNA double-strand breaks across the genome is necessary to initiate meiotic recombination at the beginning of meiotic prophase I [70,71]. *REC114* is also involved in the formation of DNA double-strand breaks that initiate meiotic recombination, and mutations in *REC114* are responsible for human multiple pronuclei formation and early embryonic arrest, indicating the gene’s critical role in oocyte meiosis and female fertility [72]. 

*BRCA2* is the principal DNA double-strand break repair gene for homologous recombination. Its lower expression in humans is significantly associated with primary ovarian insufficiency, characterized by the loss of ovarian follicles and oocytes [44]. The protein complex of *SPATA22* and meiosis specific with OB-fold (*MEIOB*) interacts with replication protein A (RPA) to form a highly compacted protein-coated single-stranded DNA during meiotic homologous recombination [73]. A homozygous variant of *SPATA22* is also associated with primary ovarian insufficiency [74]. Together with other cohesins, *SMC1B* participates in sister chromatid cohesion throughout the whole meiotic process [75]. *TEX12*, *SYCP1*, *SYCP2*, and *SYCE3* are the components of the synaptonemal complex, which is an evolutionarily conserved structure that holds homologous chromosomes together during the pachytene stage of meiotic prophase I, providing the structural framework for meiotic recombination and crossover formation [20,76,77]. *HORMAD1* is located in abundance on the synaptonemal complex in the unsynapsed region; thus, it potentially functions in the synapsis surveillance system [78]. *MAD2L1* is a spindle checkpoint component and is crucial for the commitment of chromosome segregation in both mitotic and meiotic cells [79]. *CNTRL* is required for cell cycle progression and cytokinesis in mitotic cells; however, in the oocytes, it is localized to meiotic spindles and concentrated at the spindle poles and midbody, in order to play a role in regulating the asymmetric division of meiotic oocytes [80,81].

Moreover, the biological processes of eight convergent genes, namely *RBM46*, *SLBP*, *DEPDC1*, *STK31*, *GLCCI1*, *ZMAT1*, *CCDC73,* and *C18ORF63*, are not clearly understood in the germ cells/oocytes of chickens or humans; however, they may be well-studied in other species. *Rbm46* is involved in the posttranscriptional regulation of genes essential for germ cell development in zebrafishes; also, an impairment in spermatogenesis and meiosis was observed in mutant zebrafishes [82]. SLBP protein regulates the translation of several histone mRNAs in both immature and maturing oocytes of mice [83]. *Stk31* expression was detected in the embryonic gonocytes of both sexes in mice; however, the gene is dispensable for mice reproduction [84].

**Figure 3:**
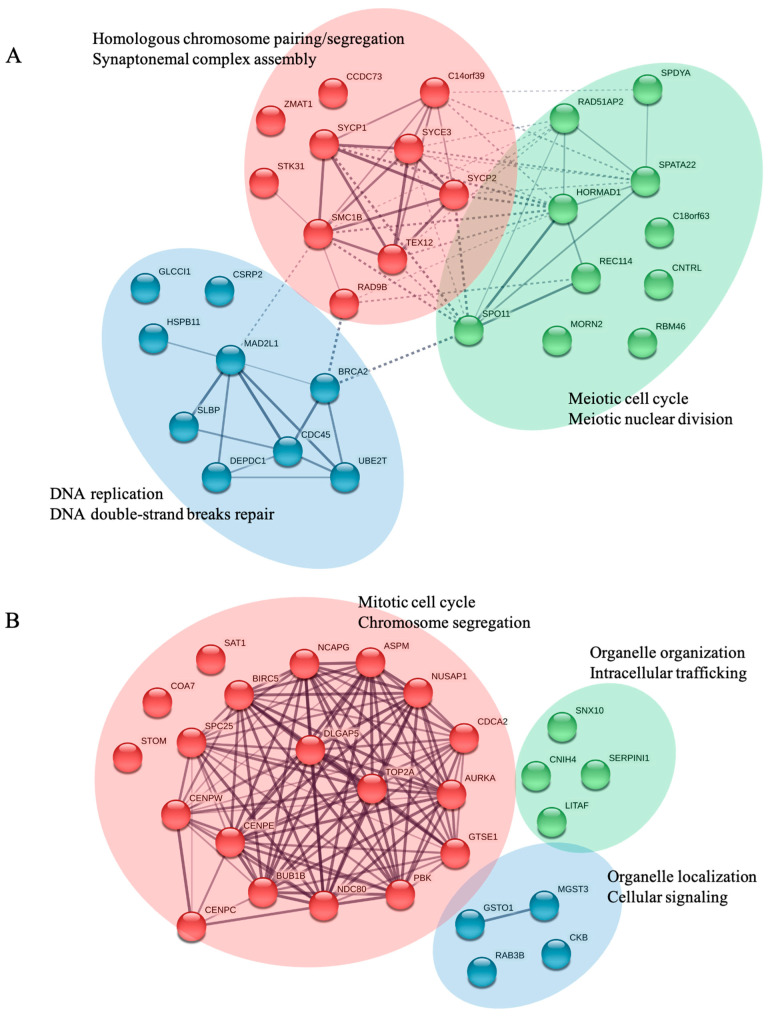
Interacting networks of the genes reviewed in this article. (**A**) Interacting networks of the chicken oocyte enriched genes that are convergent with the human oocyte. (**B**) Interacting networks of the chicken oocyte enriched genes that are divergent from the human oocyte. Interacting genes found in clusters 1, 2, and 3 are emphasized with red, green, and blue, respectively.

In order to investigate the interacting networks of the convergent genes, we used the Search Tool for the Retrieval of Interacting Genes/Proteins (STRING, Version 11.5) database [85]. According to a medium confidence search (score 0.4) and K-means clustering, all the convergent genes were clustered into three groups. Specifically, genes related to the biological processes of homologous chromosome pairing/segregation and synaptonemal complex assembly were found in cluster 1. Genes related to the biological processes of the meiotic cell cycle and meiotic nuclear division were found in cluster 2. Genes related to the biological processes of DNA replication and DNA double-strand break repair were found in cluster 3 (Figure 3A). 

## 7. Chicken Oocyte Expressed Genes Divergent from the Human Oocyte

As shown in an earlier study [25], about 27 genes were enriched in chicken oocytes at E16—1 week but showed divergent expression patterns with human oocytes (Table 1). Here, the human cells showing enriched expression of those 27 genes are considered mitotic stage female germ cells, described beginning in week 5 by Li et al. [63]. Similar to the procedure described above, the biological processes of all the divergent genes were observed using g:Profiler. Among the divergent genes, at least 21 genes, namely *ASPM*, *NUSAP1*, *CENPE*, *TOP2A*, *BUB1B*, *BIRC5*, *SPC25*, *PBK*, *DLGAP5*, *GTSE1*, *NCAPG*, *CDCA2*, *CENPW*, *AURKA*, *NDC80*, *STOM*, *SNX10*, *RAB3B*, *GSTO1*, *CNIH4,* and *CENPC*, were repeatedly identified in several mitosis-related biological processes, as well as a few meiosis-related biological processes (Figure 2B). Most of the top biological processes were related to mitotic events, such as the mitotic cell cycle, chromosome segregation, cell division, organelle fission, and mitotic nuclear division.

Before entering into meiosis, male and female germ cells undergo several rounds of mitotic cell division to increase their cell population because the initially specified cells are only a few in number. Moreover, maintaining an adequate number of germ cells is necessary for successful reproductive life in both males and females because apoptosis is a common phenomenon in germ cells. Apoptosis typically occurs in male and female germ cells that show abnormal migration to the gonads and defects during the meiotic cell cycle [86,87]. Additionally, a large proportion of oocytes undergo apoptosis during the breakdown of germ-cell cysts and the formation of primordial follicles [88]. With respect to the above divergent genes, *ASPM* encodes a mitotic spindle pole-associated protein and participates in spindle organization, spindle positioning, and cytokinesis in all dividing cells [89]. *NUSAP1* is involved in spindle microtubule organization, with selective expression in proliferating cells, and it peaks at the transition of G2 to mitosis [90]. *DLGAP5* is part of a multicomponent complex that affects the growth or stability of spindle microtubules and is required for spindle microtubule organization [91]. As promising biomarkers, both *NUSAP1* and *DLGAP5* are highly expressed and involved in the proliferation, migration, and invasion of multiple cancer cells, including ovarian cancer cells [92,93]. *GTSE1* tunes microtubule stability in mitosis to ensure chromosome alignment and segregation by suppressing MCAK (mitotic centromere-associated kinesin) microtubule depolymerase activity [94].

*CENPE* is one of the mitotic spindle assembly checkpoint components localized to kinetochores. In addition, it is required for the prevention of premature advancement to the anaphase in the presence of unattached kinetochores [95]. *BUB1B* is also a mitotic spindle assembly checkpoint component; however, it plays an important role in the kinetochore association of other spindle checkpoint proteins [96]. To form a kinetochore, which is essential for proper chromosome segregation during mitosis, constitutive centromere-associated network (CCAN) proteins are assembled on the centromere chromatin that comprises the centromere-specific histone *CENPA* [97]. *CENPC* is a CCAN protein and directly interacts with *CENPA* to nucleate the kinetochore structure [97]. Furthermore, *CENPW*, an interacting partner of *CENPT*, is also a component of the centromeric complex required for proper chromosome segregation during mitosis [98]. The NDC80 complex, which consists of *NDC80*, *SPC25,* and two other members, contributes to the microtubule–kinetochore attachment and spindle assembly checkpoint in mitosis. *SPC25* is additionally required for chromosome alignment, spindle formation, and proper spindle checkpoint signaling during oocyte meiosis [99]. *AURKA* is a member of the aurora family of kinases, which consists of *AURKA*, *AURKB,* and *AURKC.* They exhibit different subcellular localization; however, they work together to execute cell division successfully [100]. *AURKA* is mainly involved in bipolar spindle formation and chromosome alignment in mitosis and meiosis. *AURKB* is a chromosome-localized protein in mitosis and meiosis and is involved in multiple processes during cell division. *AURKC* is a chromosome- and spindle pole-localized protein expressed exclusively in meiosis [101].

*NCAPG* is one of the non-SMC (structural maintenance of chromosomes) subunits of condensin I, which play an important role in the condensation and segregation of chromosomes during mitosis [102]. Protein phosphatase 1 (PP1) is a major serine/threonine protein phosphatase involved in a wide range of cellular processes, including the condensation and segregation of chromosomes during mitosis, and it was reported that *CDCA2* selectively recruits PP1γ isoform onto mitotic chromatin in the anaphase and into the following interphase [103,104]. *PBK* is a mitotic serine/threonine protein kinase overexpressed in various actively proliferative normal cells as well as malignant tumor cells; thus, it may be a potential therapeutic target in various malignant tumors [105]. The chromatin remodeling-related protein-coding gene *TOP2A* was reported to be a major factor in the reprogramming properties of oocytes and human embryonic stem cells (ESCs) [106]. *BIRC5* regulates cell division, inhibits apoptosis in most cancer cells, and plays a role in normal physiological events, including oogenesis and embryogenesis [107]. *STOM* is a ubiquitously expressed gene, and its product is an oligomeric, monotopic membrane protein associated with cholesterol-rich membranes [108]. *SNX10* is a member of the sorting nexin protein families that possess a PX (phox homology) domain; it is responsible for membrane attachment to organelles of the secretory and endocytic systems via the binding of phosphoinositide lipids. Particularly, *SNX10* enhances the fusion of Golgi-derived vesicles with endosomes [109]. *CNIH4* is an endoplasmic reticulum (ER)/ER–Golgi intermediate complex (ER/ERGIC) localized protein, which promotes the exit of G protein-coupled receptors (GPCRs) from the early secretory pathway, most likely through interactions with the COPII component proteins [110]. *RAB3B* is one of the members of the Rab family. It exhibits GTPase activities and acts as a central regulator of vesicular traffic [111,112]. *RAB3B* expression has been associated with several cancer cells, where the silencing of *RAB3B* significantly inhibits cell proliferation and promotes apoptosis [112,113]. *GSTO2* was detected ubiquitously; however, it was localized in the cytoplasm only or both the cytoplasm and the nucleus in a cell-specific manner. *GSTO2* plays an important role in cellular signaling [114].

Moreover, the divergent genes for which the germ cell/oocyte-related biological processes are not clearly known in chickens or humans are *SAT1*, *CKB*, *MGST3*, *COA7*, *LITAF*, and *SERPINI1*. The role of *sat1* as a membrane transporter (sulphate transporter) was reported in the oocytes of amphibians [115]. *CKB*, which encodes the enzyme creatine kinase, was implicated in sperm quality and maturity in men with oligospermia [116]. The expression level of *LITAF* may control sperm survival in hen oviducts as higher levels of it in the vagina lead to sperm degradation and elimination, while lower levels in the utero–vaginal junction permit sperm survival in sperm storage tubules [117].

The interacting networks of the divergent genes were also investigated using the STRING database. According to a medium confidence search (score 0.4) and K-means clustering, most of the genes related to the biological processes of the mitotic cell cycle and chromosome segregation were strongly interactive and found in cluster 1. In contrast, most of the divergent genes related to the biological processes of organelle organization and intracellular trafficking, which were found in cluster 2, were not interactive. Similarly, the divergent genes related to the biological processes of organelle localization and cellular signaling, which were found in cluster 3, were poorly interactive (Figure 3B).

## 8. Conclusions

This review reports that germ cell specification and migration before settlement in the gonads and ovarian follicular development and maturation after sexual maturity exhibit species-specific events when comparing chickens and humans. Furthermore, the middle stages of development, including the differentiation of oogonia into primary oocytes and the formation of primary and secondary follicles, are essentially similar between chickens and humans. Therefore, genes critically involved in oocyte meiosis, such as those involved in homologous chromosome pairing and segregation, synaptonemal complex assembly, DNA replication, double-strand break repair, meiotic nuclear division, etc., are convergent between chickens and humans. Germ cell specification and the migratory route of the early mitotic germ cells in humans differ from what is observed in chickens. This may be a reason for a set of germ-cell-specific genes that shows divergent expression and functions between humans and chickens.

## Figures and Tables

**Table 1:** Chicken oocyte enriched genes that showed convergent and divergent expression patterns with human female fetal germ cells (FGCs).

Gene	Description	Enriched in Chicken	Enriched in Human	Chromosome in Chicken	Chromosome in Human
Convergent genes					
*CSRP2*	Cysteine and glycine rich protein 2	Oocytes	Oocytes	Chr.1	Chr.12
*SMC1B*	Structural maintenance of chromosomes 1B	Oocytes	Oocytes	Chr.1	Chr.22
*HORMAD1*	HORMA domain containing 1	Oocytes	Oocytes	Chr.25	Chr.1
*RBM46*	RNA binding motif protein 46	Oocytes	Oocytes	Chr.4	Chr.4
*SLBP*	Stem-loop binding protein	Oocytes	Oocytes	Chr.4	Chr.4
*DEPDC1*	DEP domain containing 1	Oocytes	Oocytes	Chr.8	Chr.1
*CDC45*	Cell division cycle 45	Oocytes	Oocytes	Chr.15	Chr.22
*MAD2L1*	Mitotic arrest deficient 2 like 1	Oocytes	Oocytes	Chr.4	Chr.4
*UBE2T*	Ubiquitin conjugating enzyme E2 T	Oocytes	Oocytes	Chr.26	Chr.1
*BRCA2*	BRCA2, DNA repair associated	Oocytes	Oocytes	Chr.1	Chr.13
*STK31*	Serine/threonine kinase 31	Oocytes	Oocytes	Chr.2	Chr.7
*GLCCI1*	Glucocorticoid induced 1	Oocytes	Oocytes	Chr.2	Chr.7
*ZMAT1*	Zinc finger matrin-type 1	Oocytes	Oocytes	Chr.4	Chr.X
*MORN2*	MORN repeat containing 2	Oocytes	Oocytes	Chr.3	Chr.2
*HSPB11*	Heat shock protein family B (small) member 11	Oocytes	Oocytes	Chr.8	Chr.1
*TEX12*	Testis expressed 12	Oocytes	Oocytes	Chr.24	Chr.11
*C14ORF39*	Chromosome 14 open reading frame 39	Oocytes	Oocytes	Chr.5	Chr.14
*RAD51AP2*	RAD51 associated protein 2	Oocytes	Oocytes	Chr.3	Chr.2
*SPATA22*	Spermatogenesis associated 22	Oocytes	Oocytes	Chr.19	Chr.17
*CCDC73*	Coiled-coil domain containing 73	Oocytes	Oocytes	Chr.5	Chr.11
*SYCP1*	Synaptonemal complex protein 1	Oocytes	Oocytes	Chr.26	Chr.1
*SYCP2*	Synaptonemal complex protein 2	Oocytes	Oocytes	Chr.20	Chr.20
*SYCE3*	Synaptonemal complex central element protein 3	Oocytes	Oocytes	Chr.1	Chr.22
*C18ORF63*	Chromosome 18 open reading frame 63	Oocytes	Oocytes	Chr.2	Chr.18
*SPDYA*	Speedy/RINGO cell cycle regulator family member A	Oocytes	Oocytes	Chr.3	Chr.2
*CNTRL*	Centriolin	Oocytes	Oocytes	Chr.17	Chr.9
*REC114*	REC114 meiotic recombination protein	Oocytes	Oocytes	Chr.10	Chr.15
*SPO11*	SPO11, initiator of meiotic double stranded breaks	Oocytes	Oocytes	Chr.20	Chr.20
*RAD9B*	RAD9 checkpoint clamp component B	Oocytes	Oocytes	Chr.15	Chr.12
Divergent genes					
*SAT1*	Spermidine/spermine N1-acetyltransferase 1	Oocytes	Mitotic GCs	Chr.1	Chr.X
*CKB*	Creatine kinase B	Oocytes	Mitotic GCs	Chr.5	Chr.14
*ASPM*	Abnormal spindle microtubule assembly	Oocytes	Mitotic GCs	Chr.8	Chr.1
*NUSAP1*	Nucleolar and spindle associated protein 1	Oocytes	Mitotic GCs	Chr.5	Chr.15
*CENPE*	Centromere protein E	Oocytes	Mitotic GCs	Chr.4	Chr.4
*TOP2A*	Topoisomerase (DNA) II alpha	Oocytes	Mitotic GCs	Chr.27	Chr.17
*BUB1B*	BUB1 mitotic checkpoint serine/threonine kinase B	Oocytes	Mitotic GCs	Chr.5	Chr.15
*BIRC5*	Baculoviral IAP repeat containing 5	Oocytes	Mitotic GCs	Chr.3	Chr.17
*SPC25*	SPC25, NDC80 kinetochore complex component	Oocytes	Mitotic GCs	Chr.7	Chr.2
*PBK*	PDZ binding kinase	Oocytes	Mitotic GCs	Chr.3	Chr.8
*DLGAP5*	DLG associated protein	Oocytes	Mitotic GCs	Chr.5	Chr.14
*GTSE1*	G2 and S-phase expressed 1	Oocytes	Mitotic GCs	Chr.1	Chr.22
*NCAPG*	Non-SMC condensin I complex subunit G	Oocytes	Mitotic GCs	Chr.4	Chr.4
*CDCA2*	Cell division cycle associated 2	Oocytes	Mitotic GCs	Chr.22	Chr.8
*CENPW*	Centromere protein W	Oocytes	Mitotic GCs	Chr.3	Chr.6
*AURKA*	Aurora kinase A	Oocytes	Mitotic GCs	Chr.20	Chr.20
*NDC80*	NDC80, kinetochore complex component	Oocytes	Mitotic GCs	Chr.2	Chr.18
*STOM*	Stomatin	Oocytes	Mitotic GCs	Chr.17	Chr.9
*MGST3*	Microsomal glutathione S-transferase 3	Oocytes	Mitotic GCs	Chr.8	Chr.1
*SNX10*	Sorting nexin 10	Oocytes	Mitotic GCs	Chr.2	Chr.7
*COA7*	Cytochrome c oxidase assembly factor 7	Oocytes	Mitotic GCs	Chr.8	Chr.1
*RAB3B*	RAB3B, member RAS oncogene family	Oocytes	Mitotic GCs	Chr.8	Chr.1
*LITAF*	Lipopolysaccharide induced TNF factor	Oocytes	Mitotic GCs	Chr.14	Chr.16
*GSTO1*	Glutathione S-transferase omega 1	Oocytes	Mitotic GCs	Chr.6	Chr.10
*SERPINI1*	Serpin family I member 1	Oocytes	Mitotic GCs	Chr.9	Chr.3
*CNIH4*	Cornichon family AMPA receptor auxiliary protein 4	Oocytes	Mitotic GCs	Chr.3	Chr.1
*CENPC*	Centromere protein C	Oocytes	Mitotic GCs	Chr.4	Chr.4

## Data Availability

The single-cell RNA sequencing data of chicken oocytes are available in the SRA database under the accession code PRJNA761874. The transcripts per kilobase million count tables of the human female FGCs were downloaded from the NCBI GEO database (GSE86146) and reanalyzed for the present paper.
